# Role of nuclear receptor PXR in immune cells and inflammatory diseases

**DOI:** 10.3389/fimmu.2022.969399

**Published:** 2022-09-02

**Authors:** Le Sun, Zhenzhen Sun, Qian Wang, Yue Zhang, Zhanjun Jia

**Affiliations:** ^1^ Nanjing Key Laboratory of Pediatrics, Children’s Hospital of Nanjing Medical University, Nanjing, China; ^2^ Jiangsu Key Laboratory of Pediatrics, Nanjing Medical University, Nanjing, China; ^3^ Department of Nephrology, Children’s Hospital of Nanjing Medical University, Nanjing, China

**Keywords:** PXR, immune cells, immune response, inflammation, disease

## Abstract

Pregnane X receptor (PXR, NR1I2), a prototypical member of the nuclear receptor superfamily, has been implicated in various processes including metabolism, immune response, and inflammation. The immune system is made up of many interdependent parts, including lymphoid organs, cells, and cytokines, which play important roles in identifying, repelling, and eliminating pathogens and other foreign chemicals. An impaired immune system could contribute to various physical dysfunction, including severe infections, allergic diseases, autoimmune disorders, and other inflammatory diseases. Recent studies revealed the involvement of PXR in the pathogenesis of immune disorders and inflammatory responses. Thus, the aim of this work is to review and discuss the advances in research associated with PXR on immunity and inflammatory diseases and to provide insights into the development of therapeutic interventions of immune disorders and inflammatory diseases by targeting PXR.

## Introduction

Pregnane X receptor (PXR) is a member of the nuclear receptor superfamily with highly conserved structures, that have an activation domain (AF), a central DNA-binding domain (DBD), a hinge region, a ligand-binding domain (LBD), and an activation function 2 (AF2) (1). Numerous studies have revealed human PXR to be commonly expressed in the small intestine, colon, and liver (2), with a small amount of expression in CD4+ and CD8+ T lymphocytes, CD19+ B lymphocytes, and CD14+ monocytes (3). It is widely reported that PXR undergoes conformational changes when an array of ligands, ranging from exogenous clinical medications to endogenous active compounds (4), bind to its LBD. The conformational changes of PXR facilitate the recruitment of coactivators and the dissociation of corepressors, leading to the subsequent initiation of PXR-targeted-gene transcription (5). It is known that PXR predominantly regulates the expression of genes related to drug transport and clearance (4, 6, 7).

Generally, human bodies defend against xenobiotic substances through both the innate response and acquired immunity (8, 9). Innate immunity consists of physiological barriers and innate immune cells such as macrophages, granulocytes, and natural killer cells, while acquired immunity involves immune organs, T and B lymphocytes, and T/B cell-derived molecules (10, 11). Despite the differences in modes of action, the two types of immune responses coordinate with each other to protect the body from pathogenic invasion through xenobiotic identification, external chemical repulsion, and noxious substance elimination (12, 13). It has been demonstrated that crosstalk between PXR and several innate immune signalings such as NF-κB, TLRs, and inflammasomes contributed to PXR-modulated innate immune responses, which further facilitated the adaptative immune responses to infections caused by microbes such as bacteria (14–16), viruses (17, 18), fungi (19, 20), and parasites (21, 22).

Biopsies from patients with immune-associated diseases as well as animal research with PXR ligands suggest a clear correlation between the biology of PXR and pathology of immune disorders and inflammation (23–31); however, the precise mechanisms are still unknown. Our document not only reviews the relationship between PXR and the immune system, but also summarizes the potential mechanisms through which PXR regulates immune disorders and inflammatory diseases, which will provide guidance for the development of therapeutic interventions targeting PXR and for the advancement of further investigations on PXR.

## PXR in immune cells

**Figure 1:**
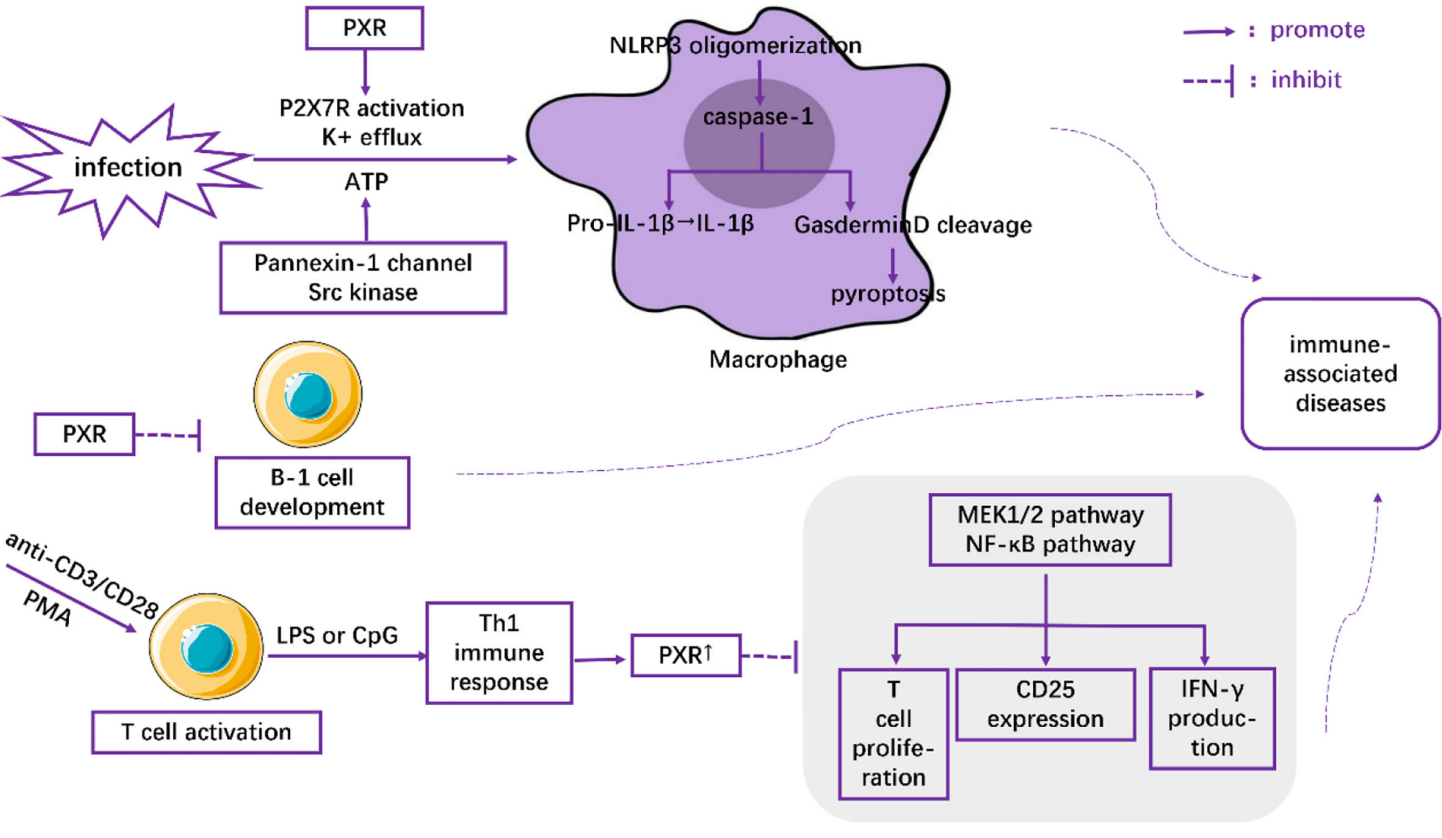
The role of PXR in the regulation of immune cells.

Macrophages play critical roles in regulating immunity and inflammation. Recent research indicates a pivotal role of PXR in macrophages by regulating NLRP3 inflammasome activation. Using rodent-specific PXR agonist PCN, Hudson and his colleagues observed that up-regulation of PXR induced the secretion of IL-1β from primed mouse macrophages, which was abrogated in PCN-treated primed macrophages isolated from PXR-/- mice (32). They also reported that PXR promoted the oligomerization of NLRP3 within activated macrophages under conditions of infection or cellular dysfunction through the stimulation of P2X7 receptor and the efflux of K+ *via* Pannexin-1 channel-and-Src kinase-dependent ATP release. Following NLRP3 oligomerization, the activation of caspase-1 subsequently induces Gasdermin D cleavage and IL-1β release (33). Cleaved Gasdermin D is reported to have the ability to punch holes in cell membranes (34, 35), which is an underlying prerequisite for pyroptosis (35–37) (**Figure 1**). Therefore, PXR induced NLRP3 inflammasome activation in activated macrophages might be associated with a large number of inflammatory diseases, such as inflammatory bowel disease (38), atherosclerosis (39), and allergic contact hypersensitivity (40). Moreover, the increased expression of PXR in high-fat diet-treated mice (41), as well as the high levels of NLRP3 inflammasome in macrophages in NAFLD (42), suggested a feedback loop in which NAFLD microenvironments upregulated the expression of PXR and its downstream activation of NLRP3 inflammasome in macrophages, aggravating the severity of NAFLD.

PXR has also been demonstrated to inhibit T cell immunity. Using anti-CD3/CD28 or PMA to activate T lymphocytes and LPS or CpG to induce Th1 immune response, Dubrac, et al. (3) found that Th1 immune response (as opposed to Th2 immune response) contributed to the expression of PXR. The overexpressed PXR, in turn, blocked the proliferation signals of T lymphocytes in a PXR-dependent fashion through inhibition of the MEK1/2 and NF-κB pathways (3). Moreover, the inhibitory effects of PXR agonists on CD25 expression and IFN-γ production were observed both in mice and human T lymphocytes (3) (**Figure 1**). All these effects were abolished when PXR was deficient (3). Thus, the inhibitory role of PXR in restraining T cell immunity might shed light on novel therapeutic strategies for inflammatory diseases.

Casey, et al. (43) found that PXR and its target genes perturbed the development and function of B-1 lymphocytes in the fetal liver (**Figure 1**). They showed that the number of B-1 cells as well as the immune capacity of neonates was reduced after their exposure to PXR agonists during the fetal period. In contrast, gestational exposure to PXR antagonists could elevate the level of B cells (43). Together, these results highlight the pivotal role of PXR as a modulator of the immune response.

## PXR in inflammatory diseases

### PXR and IBD

Inflammatory bowel diseases (IBD), including ulcerative colitis, and Crohn’s disease, are chronic relapsing disorders characterized by dysregulation of intestinal homeostasis, disruption of intestinal epithelial barriers, and an increase of intestinal permeability (44). IBD was previously regarded as a disease that mainly occurred in western populations. However, recent studies have reported that its prevalence is rising rapidly and it has become increasingly life-threatening worldwide (45). A large group of investigations has revealed the crucial role of PXR in the pathogenesis of IBD.

**Figure 2:**
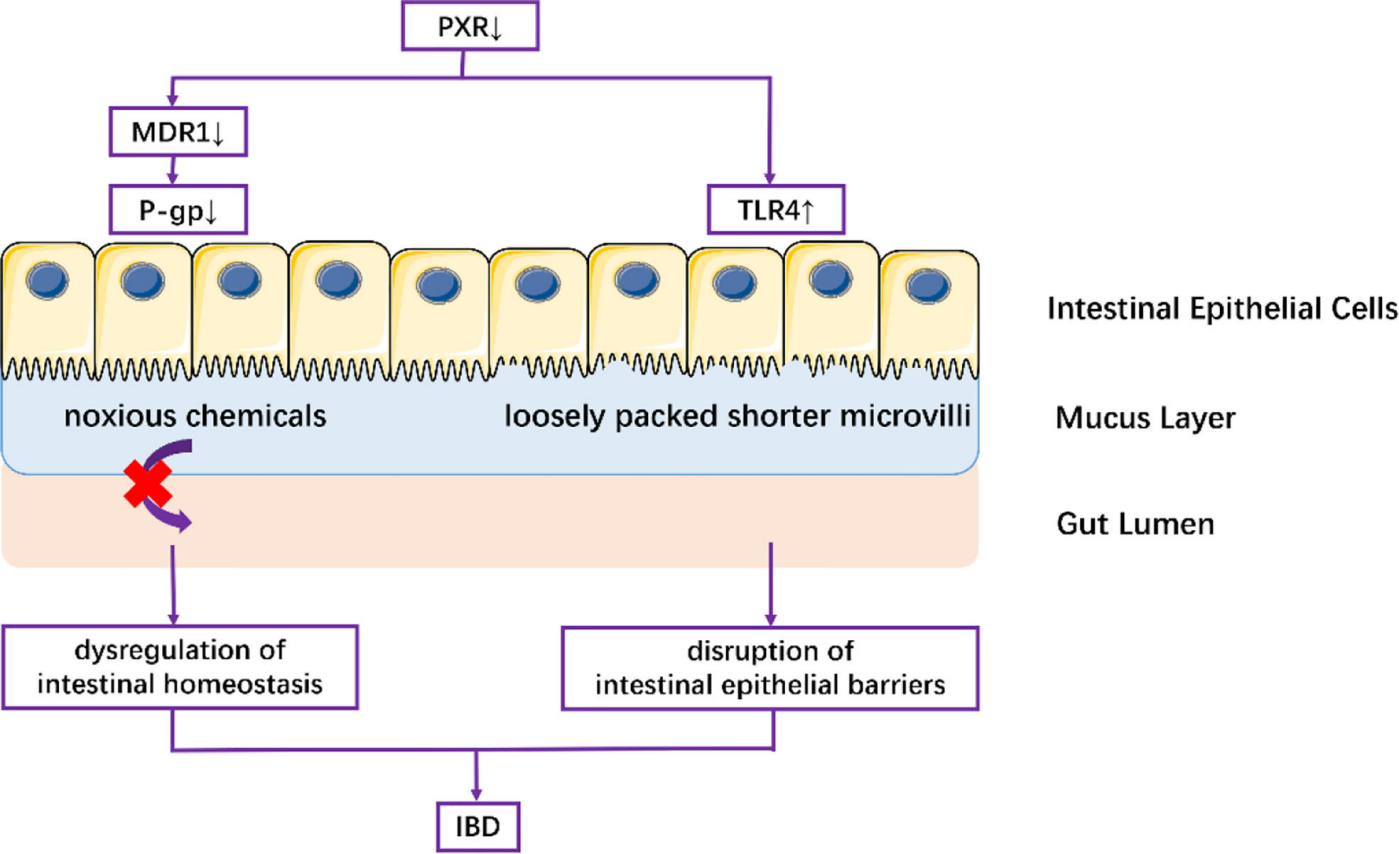
Mechanisms underlying the role of PXR in the pathogenesis of IBD.

PXR has been demonstrated to serve as a modulator to maintain intestinal homeostasis. Multidrug resistance gene (MDR) 1, mainly regulated by PXR and highly expressed in intestinal epithelial cells, has been categorized as a transporter of the ABC family (46). MDR1 regulates the expression of transmembrane protein P-glycoprotein (P-gp), which drives out toxins from the mucus layer to the gut lumen in the presence of ATP, thus reducing the accumulation of multidrug-resistant drugs in cells and maintaining intestinal homeostasis (46, 47). It has been reported that reduced P-gp expression, attributable to Mdr1a gene polymorphism, was observed in both patients with IBD and mouse models with spontaneous colitis (48); and this effect was ameliorated by rifaximin. Incubating three different intestinal model cell lines (Caco2, HT-29, and LS174T) with various concentrations of human recombinant TNF-α, Langman et al. showed that the downregulation of PXR might be the most likely cause of the downstream reduction of Mdr1a expression under conditions of intestinal inflammation (49) (**Figure 2**). Evidence from previous study has indicated that IL-1β decreased the expression and functionality of P-gp in the human intestinal epithelial cell line Caco-2 (50). Down-regulation of PXR by imidacloprid (IMI) treatment exhibited high levels of TNF-α and IL-1β both *in vivo* and *in vitro* (51).. In addition, previous work also showed that PCN prevented dextran sulfate sodium (DSS) -induced colitis through inhibition of TNF-α and IL-1β in a PXR-dependent manner (52), suggesting a negative correlation between PXR and IL-1 in modulating intestinal homeostasis under the context of IBD. Taken together, PXR reduction in inflammatory intestinal epithelial cells decreases the expression of the Mdr1a gene and downstream P-gp, contributing to dysregulated intestinal homeostasis.

PXR deficiency is the preliminary driving force for disrupted intestinal epithelial barriers and increased intestinal permeability in IBD. In one study, Nr1i2-/- mice exhibited enhanced ultrastructural defects as characterized by a significant reduction in villus-crypt ratio, loosely packed shorter microvilli, and increased electron density of tight-junction (Tj) and adherens-junction (Aj) complexes in the intestinal epithelium (28). These pathological changes were significantly ameliorated in Nr1i2+/+ mice. Furthermore, the Nr1i2-/- mice also exhibited enhanced intestinal permeability as confirmed by the researchers’ finding that the change of mean levels of FITC-dextran in the serum of Nr1i2-/- mice (488.9%) was nearly three times larger than that of Nr1i2+/+ mice (193%). Their results suggested that PXR deficiency exacerbated the disruption of intestinal barrier function. The mechanism underlying the effect of PXR on intestinal barrier and permeability is likely through TLR4. A negative-related expression of PXR and Tlr4 mRNA was observed both in Caco-2 cells and primary human intestinal mucosa. It was observed that inhibition of TLR4 (as opposed to TLR2) in Nr1i2-/- mice could decrease TNF-α and IL-6 levels, which is consistent with the result that Pxr-/-Tlr4-/- mice (compared with Pxr-/-Tlr2-/- mice) could effectively protect against Listeria monocytogenes infection (53) (**Figure 2**). Therefore, TLR4 plays a central role in PXR deficiency-mediated impaired intestinal epithelial barriers and permeability.

It is universally recognized that PXR can inhibit NF-κB pathway-related inflammatory responses in a ligand-dependent manner (54, 55). Moreover, several effective PXR agonists such as rifaximin (25, 32), curcumin (56), and Solomonterol A (29, 57–59), which have extended our understanding of the anti-inflammatory role of PXR, are expected to be leveraged as potential therapeutic drugs for clinical treatment of IBD.

In conclusion, the effect of PXR on IBD pathogenesis is sophisticated and more investigations are needed to further illuminate its novel functions and potential molecular mechanisms.

### PXR and atherosclerosis

Atherosclerosis is considered a chronic inflammatory disease of blood vessel walls. The main lesions are located in the subendothelial space, where the chemokine-driven mononuclear cells recruit and differentiate into macrophages, a prerequisite for atherosclerosis (60). Macrophage and endothelial cell (ECs)-mediated proinflammatory cytokine release, as well as foam cell formation in subendothelial lesions, have close associations with the pathogenesis of atherogenesis (61). Regarded as a leading cause of vascular disease, atherosclerosis is gaining extensive attention worldwide (62).

**Figure 3:**
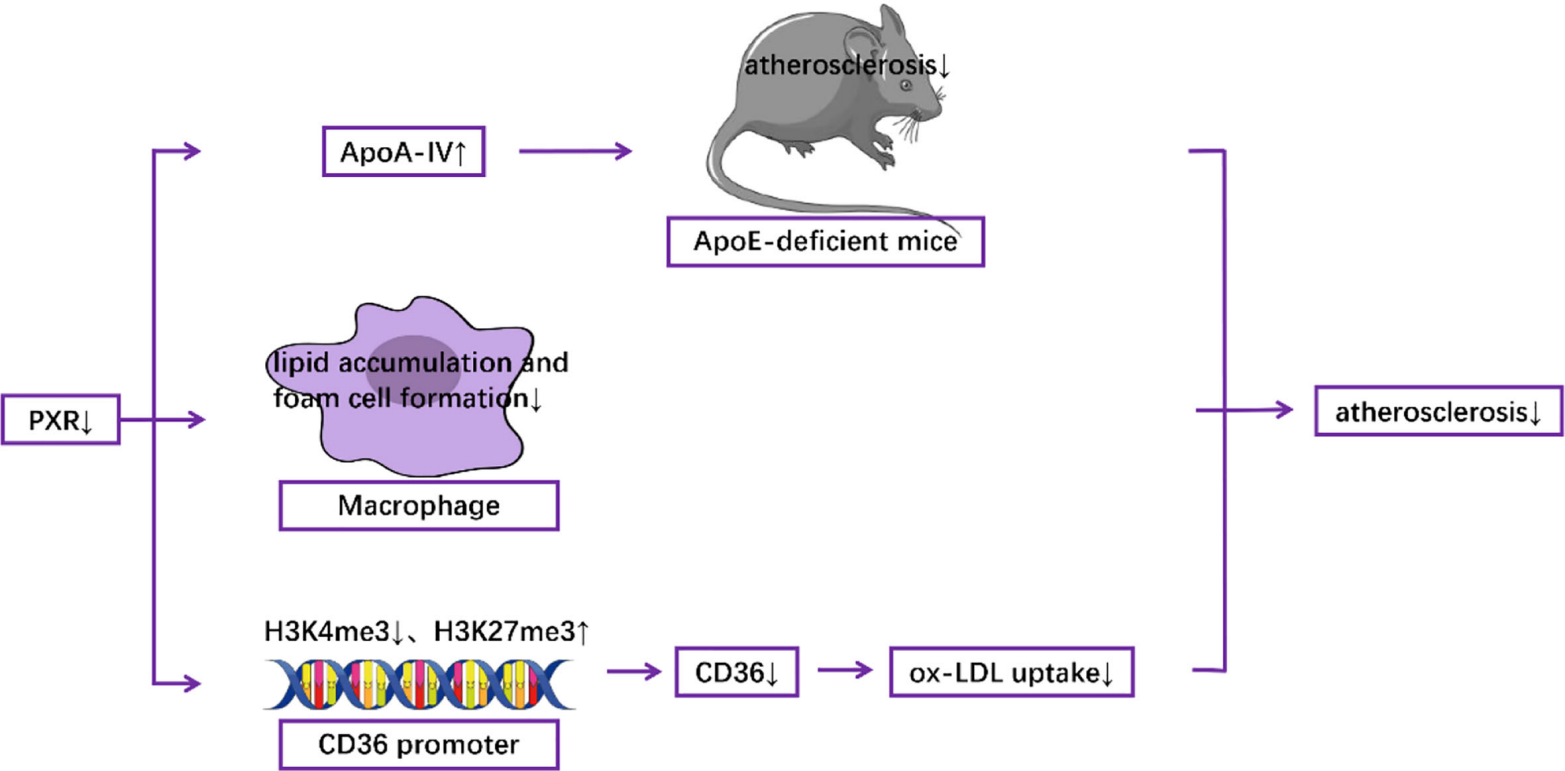
Mechanisms underlying the role of PXR in the pathogenesis of atherosclerosis.

In animal models, myeloid-specific deficiency of PXR decreased atherosclerosis in LDL receptor-deficient mice (63). In addition, Sui et al. reported that exposure to perinatal Bisphenol A, a potential agonist for human PXR, increased atherosclerosis both in adult male PXR-humanized mice (64) and PXR-humanized ApoE-deficient mice (65). Consistent with these results, Zhou et al. demonstrated that activation of PXR induced hypercholesterolemia in wild-type mice and accelerated atherosclerosis in ApoE deficient mice (66). In contrast, deficiency of PXR decreased atherosclerosis in ApoE-deficient mice (67). At the molecular level, it has been documented that the detrimental effect of PXR was mediated by decreased levels of hepatic ApoA-IV, which has anti-atherogenic properties (67). Furthermore, PXR deletion can reduce the formation of foam cells and the accumulation of lipids in macrophages (63), which contributes to atherosclerotic pathogenesis (68–73). PXR ablation also decreased the expression of CD36, a well-known scavenger receptor (63), by decreasing the levels of H3K4me3, which is responsible for gene activation, and by increasing the levels of H3K27me3, which is responsible for gene suppression, in the promoter of CD36, resulting in the reduction of ox-LDL uptake (64) (**Figure 3**). Furthermore, RNA-Seq analysis indicates that PXR ligands influenced the expression of many genes related to atherosclerosis in macrophages *in vitro*. For example, genes involved in lipid metabolism and inflammatory responses are modulated by the PXR agonist PCN (Pregnenolone-16α-carbonitrile, a rodent-specific pregnane X receptor activator) in a PXR-dependent manner (63).

Treatment with PXR ligands can inhibit platelet function, such as platelet aggregation and adhesion, and granule secretion, reduce thrombus formation (74). Additionally, the human-specific ligand, SR12813, was observed to attenuate the formation of thrombi *in vivo* in humanized PXR transgenic mice through the inhibition of Src-family kinases (SFKs) (74). It seems plausible that the anti-thrombotic effects of PXR ligands may provide additional anti-atherosclerotic properties. Although the contradictory results can be explained by the different conditions and animal models used. Because foam cells formation is the driving force behind atherosclerosis, the anti-thrombotic function of PXR may not play a decisive role in the pathologic processes of atherosclerosis.

Overall, these results suggest that antagonizing PXR activity may provide therapeutic benefits in the management of atherosclerosis.

### PXR and Non-alcoholic fatty liver disease

Non-alcoholic fatty liver disease (NAFLD), which encompasses steatosis with or without mild inflammation and non-alcoholic steatohepatitis (NASH), has a global prevalence of 25% (75). Accumulating evidence has indicated an association between PXR and the molecular mechanism of NAFLD pathogenesis.

**Figure 4:**
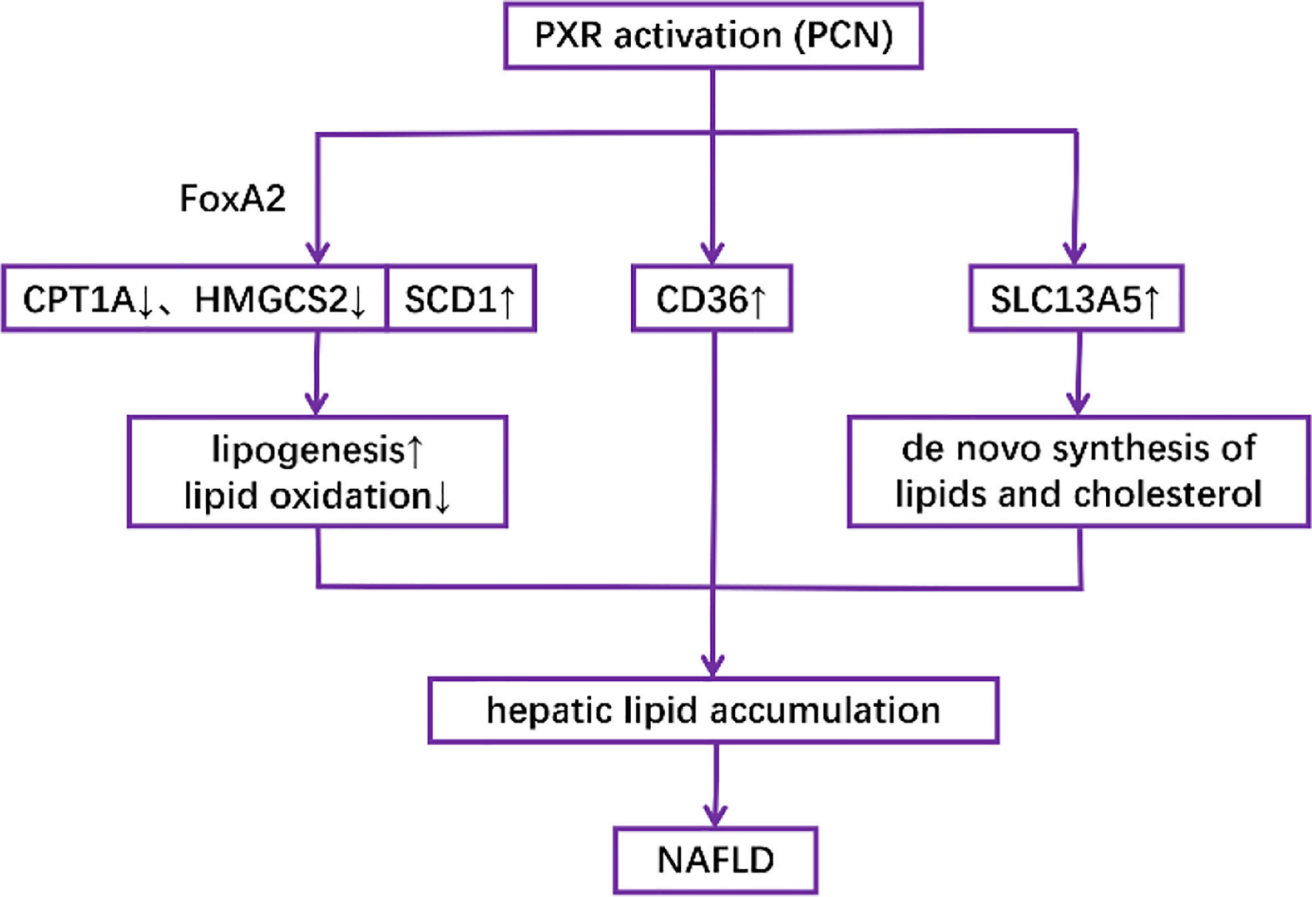
Mechanisms underlying the role of PXR activation in the pathogenesis of NAFLD.

Activation of PXR in both mouse models and human primary hepatocytes has been shown to increase lipogenesis while decreasing lipid oxidation, thus promoting the accumulation of lipids in the liver (76–79). Mechanically, it has been revealed that PCN downregulates the expression of CPT1A (β-oxidation) and mitochondrial HMGCS2 (ketogenesis) but upregulates the expression of SCD1 (lipogenesis) in a PXR-dependent manner partly through the interaction between PXR and the insulin response forkhead factor FoxA2 (77). It has also been revealed that both genetically and pharmacologically activated PXR in the liver induce fatty acid translocase (FAT)/CD36 proteins other than SREBP-1c, which is responsible for the transcription of lipogenic genes (76). Another possible mechanism is the induction of SLC13A5, which can transport circulatory citrate to the hepatocyte under the control of PXR, thus facilitating *de novo* lipogenesis (79) (**Figure 4**).

Although severe spontaneous hepatic steatosis has also been observed in PXR-/- mice (77), He et al. (80) indicated that PXR ablation protected mice from both high fat diet-induced and gene mutation-induced insulin resistance and hepatic steatosis. These contradictory findings, which have added yet another layer of complexity to our understanding of the role of PXR in lipid metabolism homeostasis and NAFLD, may be explained by different genetic backgrounds of mice used and PCN treatment dosage and schedules. Whether targeting PXR can be used as a therapeutic strategy for NAFLD and its associated diseases requires further validation.

## Others

In addition to the role of PXR in IBD, atherosclerosis, and NAFLD, PXR has also been demonstrated to act as a critical regulator in other inflammatory diseases including acute kidney injury, ascending placentitis, and atopic dermatitis. PXR has recently been found to target AKR1B7, a member of the AKR superfamily, to improve mitochondrial function and to protect against renal dysfunction in acute kidney injury (81, 82). Furthermore, Ali et al. (83) revealed significant downregulation of PXR and several other steroid hormone receptors during equine ascending placentitis in the chorioallantois and/or endometrium. Moreover, it has also been observed that lipophilic components of environmental pollutants penetrated the impaired skin barrier and activated PXR in the basal layer, inducing the release of cytokines by keratinocytes and skin T cells. Cytokines, which were derived from Th2 and Th17 cells, further mediated skin inflammatory responses similar to atopic dermatitis (24). However, no research has shown the relationship between PXR and trained immunity to date, which needs investigation in the future.

Accumulating evidence has indicated the involvement of PXR in the development of cancer. It has been described that PXR activation was protective in hepatocellular carcinoma (HCC), as evidenced by the fact that the PXR level was decreased in mice with HCC, while the migration, adhesion, and invasion were reduced in HepG2 cells transfected with PXR (84). In addition, Zsanett and collaborators reported that indolepropionic acid (IPA), a bacterial metabolite, inhibited cell growth in breast cancer through the activation of PXR and AHR receptors. And breast cancer patients with higher expression of PXR and AHR exhibited a better prognosis (85). However, it has also been revealed that PXR conferred colon cancer cells the ability to tolerate the genotoxicity of chemotherapeutic agents through MDR1/CYP3A4-mediated drug metabolisms, contributing to drug resistance (86). In conclusion, these results suggest that tissue-specific regulation of PXR may play an important role in tumor growth and the discovery of the pleiotropic role of PXR in cancers may open the door for novel approaches treating malignant tumors.

It was also revealed that the bile acid-activated receptor PXR modulated the communications between the intestinal immune system and intestinal microbiota (87). Moreover, additional studies reported the increased infiltration of inflammatory cells accompanied with the decreased expression of PXR in the liver of colitis mice treated with DSS (88). Collectively, these studies provided a preliminary hint that PXR may serve as a mediator in the “gut-liver” axis.

## Summary and future perspectives

Considered as a pivotal nuclear receptor, PXR has been reported to be involved in regulating several immune cells and modulating the process of immune responses. As mentioned above, the activation of PXR promotes the NLRP3-mediated pyroptosis, inhibits T cell proliferation, and represses the development and function of B-1 lymphocytes in the fetal liver. PXR has also been demonstrated to participate in the pathogenesis of inflammatory diseases. The sophisticated roles of PXR, such as the protective effect of PXR in IBD, the pathogenic role of PXR in atherosclerosis, and the bidirectional functions of PXR in NAFLD, provide us with a profound understanding of the immune system and immune response-associated diseases. However, the detailed mechanisms underlying the PXR-mediated pathologic processes of inflammatory diseases need to be further explored and more investigations targeting on clinical cases are needed. Our document illuminates the multiple roles of PXR in immune- and inflammation-related diseases, which not only unravels the potential molecular mechanisms of these pathologic events but also provides insights into the development of therapeutic interventions targeting PXR and for the advancement of further investigations on PXR.

## Author contributions

LS wrote the manuscript. ZS and QW revised the text. ZJ and YZ supervised the work. All authors contributed to the article and approved the submitted version.

## Funding

This work was supported by grants from the National Natural Science Foundation of China (82070701, 81873599, 82170754, 81700651) and Science and Technology Development Foundation of Nanjing Medical University (NMUB2020038).

## Conflict of interest

The authors declare that the research was conducted in the absence of any commercial or financial relationships that could be construed as a potential conflict of interest.

## Publisher’s note

All claims expressed in this article are solely those of the authors and do not necessarily represent those of their affiliated organizations, or those of the publisher, the editors and the reviewers. Any product that may be evaluated in this article, or claim that may be made by its manufacturer, is not guaranteed or endorsed by the publisher.
